# The expanding organelle lipidomes: current knowledge and challenges

**DOI:** 10.1007/s00018-023-04889-3

**Published:** 2023-08-02

**Authors:** Maria J. Sarmento, Alicia Llorente, Toni Petan, Denis Khnykin, Iuliana Popa, Matea Nikolac Perkovic, Marcela Konjevod, Morana Jaganjac

**Affiliations:** 1 Instituto de Medicina Molecular, Faculdade de Medicina, Universidade de Lisboa, 1649-028 Lisbon, Portugal; 2 Department of Molecular Cell Biology, Institute for Cancer Research, Oslo University Hospital, 0379 Oslo, Norway; 3 Department for Mechanical, Electronics and Chemical Engineering, Oslo Metropolitan University, 0167 Oslo, Norway; 4 Faculty of Medicine, Centre for Cancer Cell Reprogramming, University of Oslo, Montebello, 0379 Oslo, Norway; 5 Department of Molecular and Biomedical Sciences, Jožef Stefan Institute, Ljubljana, Slovenia; 6 Department of Pathology, Oslo University Hospital, Oslo, Norway; 7 Pharmacy Department, Bâtiment Henri Moissan, University Paris-Saclay, 17 Avenue des Sciences, 91400 Orsay, France; 8 Division of Molecular Medicine, Ruder Boskovic Institute, 10000 Zagreb, Croatia

**Keywords:** Lipidomics, Lipids, Mass spectrometry, Cellular organelles, Subcellular fractionation

## Abstract

Lipids in cell membranes and subcellular compartments play essential roles in numerous cellular processes, such as energy production, cell signaling and inflammation. A specific organelle lipidome is characterized by lipid synthesis and metabolism, intracellular trafficking, and lipid homeostasis in the organelle. Over the years, considerable effort has been directed to the identification of the lipid fingerprints of cellular organelles. However, these fingerprints are not fully characterized due to the large variety and structural complexity of lipids and the great variability in the abundance of different lipid species. The process becomes even more challenging when considering that the lipidome differs in health and disease contexts. This review summarizes the information available on the lipid composition of mammalian cell organelles, particularly the lipidome of the nucleus, mitochondrion, endoplasmic reticulum, Golgi apparatus, plasma membrane and organelles in the endocytic pathway. The lipid compositions of extracellular vesicles and lamellar bodies are also described. In addition, several examples of subcellular lipidome dynamics under physiological and pathological conditions are presented. Finally, challenges in mapping organelle lipidomes are discussed.

## Introduction

Over the years, increasing attention has been directed to the composition, organization and function of lipids in mammalian cell membranes and internal compartments. Although cell compartmentalization has been traditionally thought to be the main purpose of lipids, it is clear now that lipids exhibit functions in addition to those related to their structures. Indeed, lipids are involved in processes ranging from energy production, defensive antioxidative responses and cell signaling [1–4] to epigenetic control, temperature regulation and inflammation [5, 6]. Unsurprisingly, it has been estimated that more than 1000 different lipids compose the lipidome of a single mammalian cell [7–9]. The elucidation of the functions of individual lipid species is a challenging scientific goal due to the large variety of lipids and because the role of a particular lipid molecule is intrinsically connected to the cooperative nature and specific properties of lipid assemblies, such as bilayers. The functions of these highly dynamic structures have been largely defined by their specific lipid compositions and dynamic functional changes in response to signaling events, changes in metabolic flux, and microenvironmental conditions. Hence, lipids play pleiotropic roles but also a multitude of context-dependent specific roles.

**Fig. 1:**
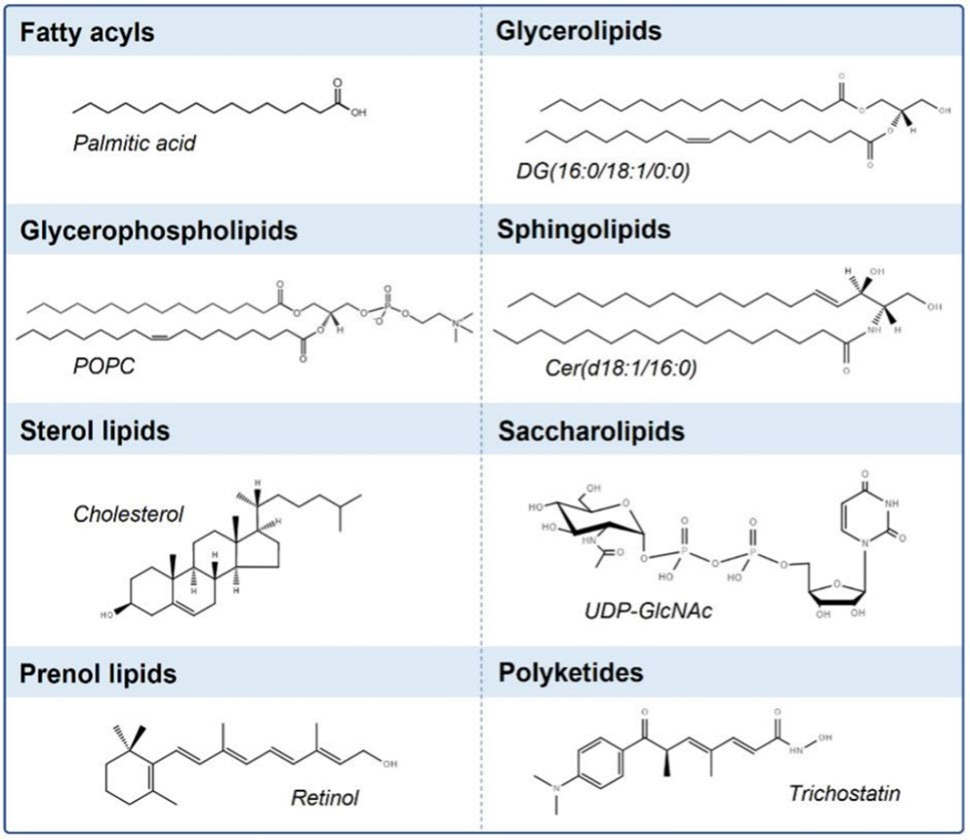
Schematic representation of the main lipid classes, including one example per class, as characterized by the LIPID MAPS consortium

**Fig. 2:**
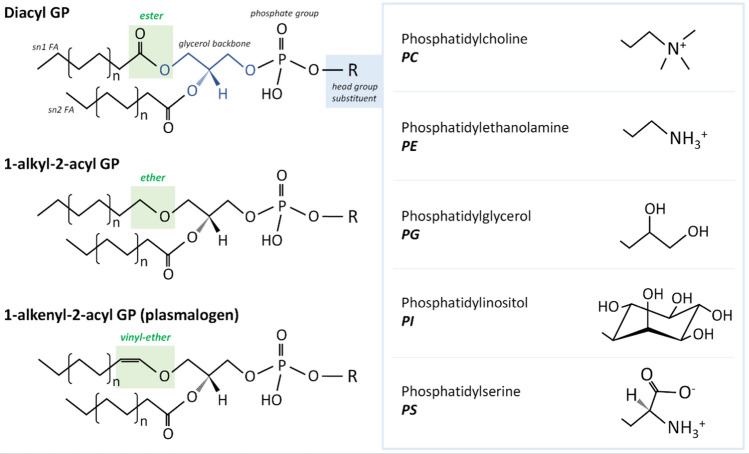
Basic structure of glycerophospholipids. Those with either ester or ether versions, as well as the most common headgroups, are presented

To address the increasing amount of data generated by the lipid research community, the LIPID MAPS consortium has been working on uniformizing both the classification and categorization of lipids [10–12]. The Consortium defines lipids as ‘hydrophobic or amphipathic small molecules that may originate entirely or in part by carbanion-based condensations of ketoacyl thioesters and/or by carbocation-based condensations of isoprene units. Depending on their structure and properties, lipids are distributed into 8 distinct categories: fatty acyls, glycerolipids, glycerophospholipids, sphingolipids, sterol lipids, prenol lipids, saccharolipids and polyketides (Fig. 1). A complete description of each of these classes is beyond the scope of this review and can be found elsewhere [10–13]. In general, mammalian cell lipids comprise mainly glycerophospholipids (Fig. 2), glycerolipids, sphingolipids and sterol lipids, with other classes represented to a lesser degree [14]. However, the specific lipid composition depends on each particular membrane/organelle. Clearly, lipid synthesis and metabolism, as well as intracellular trafficking, characterize lipid homeostasis and, thus, the specific organelle lipidome. Notably, lipids are asymmetrically distributed in the outer and inner leaflet of cellular membranes.

In addition to the intracellular variation in lipid profiles, the lipidome of mammalian cells deviates significantly across different tissues [15–17]. In mice, lipidome has been previously shown to be more similar among tissues with related functions, e.g., liver and kidney, spleen and thymus, or cardiac and skeletal muscle [16]. In all tissues, glycerophospholipids constitute the most abundant class (phosphatidylcholine (PC) > phosphatidylethanolamine (PE) > phosphatidylinositol (PI) > phosphatidylserine (PS)), although the fingerprint of specific glycerophospholipid species differs, as does that among lipid classes [18, 19]. In rat models, sphingolipids and sterol lipids are most abundant in the kidney, followed by the liver and heart, while glycerolipids are present in higher amounts in adipose tissue, followed by the kidney and liver [19]. However, more studies are needed to understand whether this abundance pattern applies to other mammals. The content of ether lipids **(**Fig. 2**)**, glycerophospholipids in which the acyl chain at the sn-1 position of the glycerol backbone is attached by an ether bond, is highest in the rat brain and heart, followed by skeletal muscle, adipose tissue, the kidney and the liver [18]. In regard to the fatty acyl chains, polyunsaturated chains have been shown to be enriched in neuronal membranes [20, 21]. Similarly, Hunt and coworkers concluded that PC molecules in the liver were highly unsaturated, and lungs were enriched in highly saturated PC species [22, 23]. Overall, despite great accomplishments over the years, the lipid fingerprint of each organelle/cell/tissue is still far from being fully characterized and remains challenging, especially when we consider the differences in human lipidomes in health and disease context. There have only been a few comparative studies that have provided information on the amount of lipids in distinct organelles; therefore, future quantitative comparative lipidomic investigations are required to gain a better understanding of subcellular lipidomes.

In this review, we present the information available about the lipid composition of cellular organelles in mammalian cells. In particular, the lipidome of the nucleus, mitochondrion, endoplasmic reticulum, Golgi apparatus, plasma membrane and organelles in the endocytic pathway are described. In addition, we also include information about cell-derived extracellular vesicles and lamellar bodies, which are lysosome-related organelles found in some specialized cells. Preparation of pure organelles is essential to obtain reliable information about their respective lipidomes, and meeting this criterion is considered a main challenge to studies aiming to profile the specific molecular profiles of organelles and is discussed in this review. Finally, as the cellular lipidome is extremely dynamic and because the lipid composition changes in response to various factors, several examples of lipidome dynamics under physiological and pathological are described.

## Lipid composition and organelle function, dynamics and integrity

**Fig. 3:**
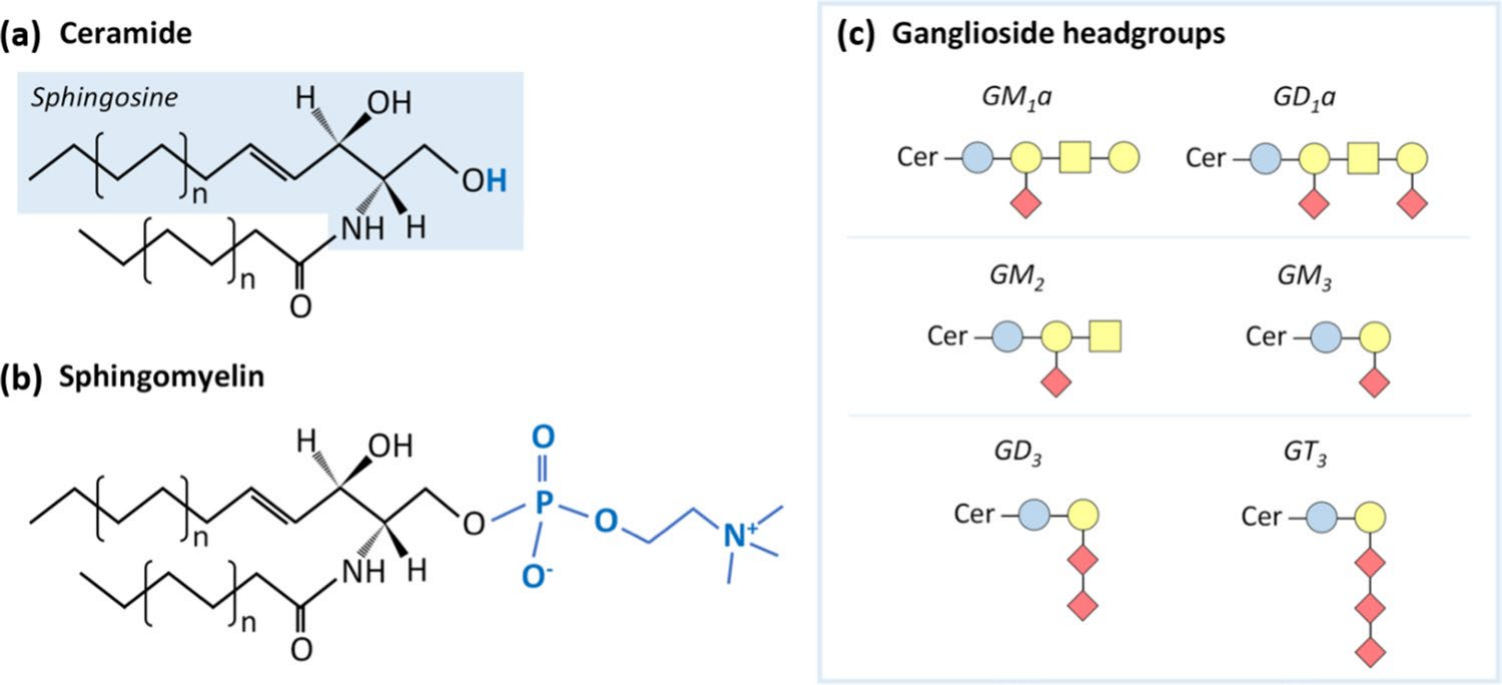
Schematic representation of different sphingolipids. **a** Ceramide is the building block of more complex sphingolipids. **b** Sphingomyelin is one of the most common sphingolipids in mammalian cells. **c** Examples of ganglioside headgroups. Gangliosides are glycosphingolipids with a ceramide backbone and headgroups with different sugar unit combinations. Blue circle: glucose; yellow circle: galactose; yellow square: N-acetylgalactosamine; and red diamond: N-acetylneuraminic acid

Most lipids are synthesized in the endoplasmic reticulum, and distal organelles such as the plasma membrane have very limited capacity to produce the lipids that form them [24, 25]. Notably, the endoplasmic reticulum produces structural phospholipids, cholesterol (Chol) and ceramide (Cer), the building blocks of more complex sphingolipids (Fig. 3) [24]. The subsequent modification and sorting of each lipid species to distant organelles are mainly realized in the secretory pathway, specifically within the Golgi apparatus. Lipid transport from the endoplasmic reticulum to the Golgi and other organelles, such as the plasma membrane, lysosomes and lipid droplets is mediated by both vesicular and nonvesicular transport mechanisms [26, 27]. The latter include direct contact of different organelles with the reticular membrane and the transfer of lipids at specific locations called membrane contact sites [28, 29]. Interestingly, the endoplasmic reticulum and the plasma membrane are the organelles that present the most difference in terms of lipid composition, with the Golgi presenting an ‘intermediate’ lipidome. In fact, although the nuclear envelope, lipid droplets and the cis-Golgi compartment share lipidomic features with the endoplasmic reticulum, the lipidome in the trans-Golgi network and the organelles in the endocytic pathway are more similar to the of the plasma membrane [4, 30]. These similarities and have contributed to the idea that eukaryotic cells are fundamentally organized into two membrane territories with different lipid compositions and distinct membrane-recycling systems with primary involvement of either the endoplasmic reticulum or plasma membrane [30–32]. In addition to this spatial organization of the cellular lipidome, all these lipid synthesis processes that are orchestrated in a timely manner in a cell, depend on the cell cycle, external stimuli, circadian rhythm and, overall, the life cycle of the organism [33–35].

**Fig. 4:**
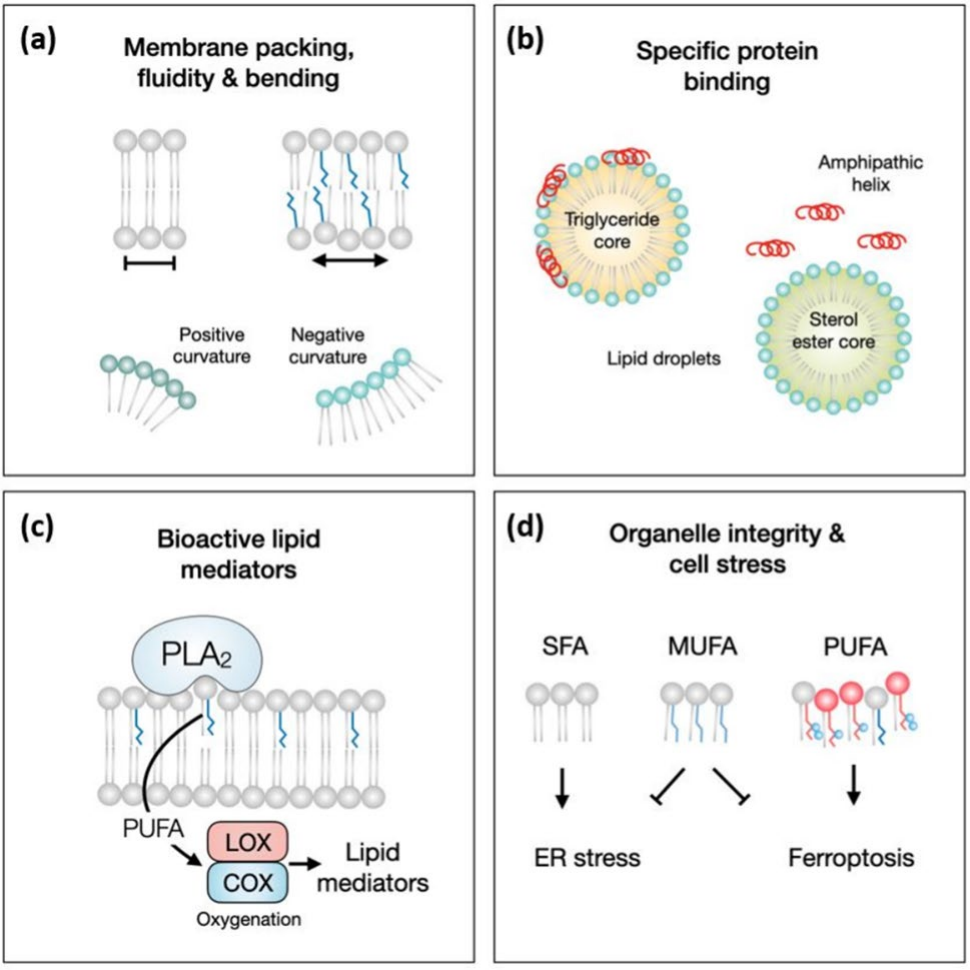
Lipid composition affects organelle function, dynamics and integrity. **a** The composition of fatty acyl chains and polar headgroups in membrane phospholipids determine biophysical membrane properties, such as packing, bending and fluidity. **b** Lipids control protein binding to organelles. The composition of the hydrophobic lipid droplet core determines the affinity of amphipathic helix-containing proteins for the organelle (not to membrane bilayers) or to subsets of cellular lipid droplets with a specific oil composition. **c** Phospholipase A_2_ (PLA_2_) enzyme releases polyunsaturated fatty acids (PUFAs) from membrane phospholipids. PUFAs are converted into bioactive lipid mediators, such as eicosanoids, via various oxygenase enzymes, including lipoxygenases (LOXs) and cyclooxygenases (COXs). **d** The proportion of saturated fatty acids (SFAs), monounsaturated fatty acids (MUFAs) and PUFAs in membrane phospholipids is crucial to membrane and organelle function and integrity. Excess SFAs may cause endoplasmic reticulum stress, and excess PUFAs can be oxidized into toxic lipid peroxides that can cause ferroptotic cell death. Both stress-producing processes are mitigated by MUFAs

Why is the cellular lipid composition important, and how does it affect organelle function? First, lipid composition determines the biophysical properties of lipid assemblies. For example, membrane phospholipid saturation depends on the relative amounts of saturated, monounsaturated, and polyunsaturated fatty acyl chains. The mobility, rigidity and size of these chains determine the bilayer fluidity and bending properties [36] (Fig. 4a), thus influencing several membrane-associated processes within a cell; these include, among others, membrane–protein interactions and protein function, the speed of signal propagation (ligands, products and substrates) in the two-dimensional plane of a membrane, and various membrane fusion and fission processes that are required for organelle synthesis and growth, tubular dynamics and vesicular transport [17, 30, 37]. The sterol content levels determines the rigidity of the plasma membrane (as well as its 2D diffusion) and directly affects the formation of raft-like domains, which act as transient platforms for the assembly of protein complexes involved in signal transduction and other processes [38]. Second, specific lipids localized at certain subcellular locations function as signaling molecules that activate enzymatic or signaling processes or as anchors for the attachment of proteins to specific membranes/organelles. Notably, in addition to polar lipids, neutral lipids can control protein binding and activity. For example, the composition of neutral lipids in the hydrophobic core of lipid droplets determines the affinity of the droplet for amphipathic helix-containing proteins to lipid droplets [39, 40] (Fig. 4b). Third, some lipids can be biochemically converted into soluble signaling molecules that act in the extracellular milieu. For example, membrane-resident polyunsaturated fatty acids released from phospholipids by phospholipase A_2_ are converted by oxygenases into a large family of bioactive signaling molecules [41] (Fig. 4c). Finally, we are only beginning to understand how lipid modifications, such as oxidation [42, 43], impair membrane and organelle function and trigger both specific and general cellular responses that contribute to membrane and cellular integrity maintenance (Fig. 4d**)** [44–46].

## Lipid composition of subcellular compartments

**Fig. 5:**
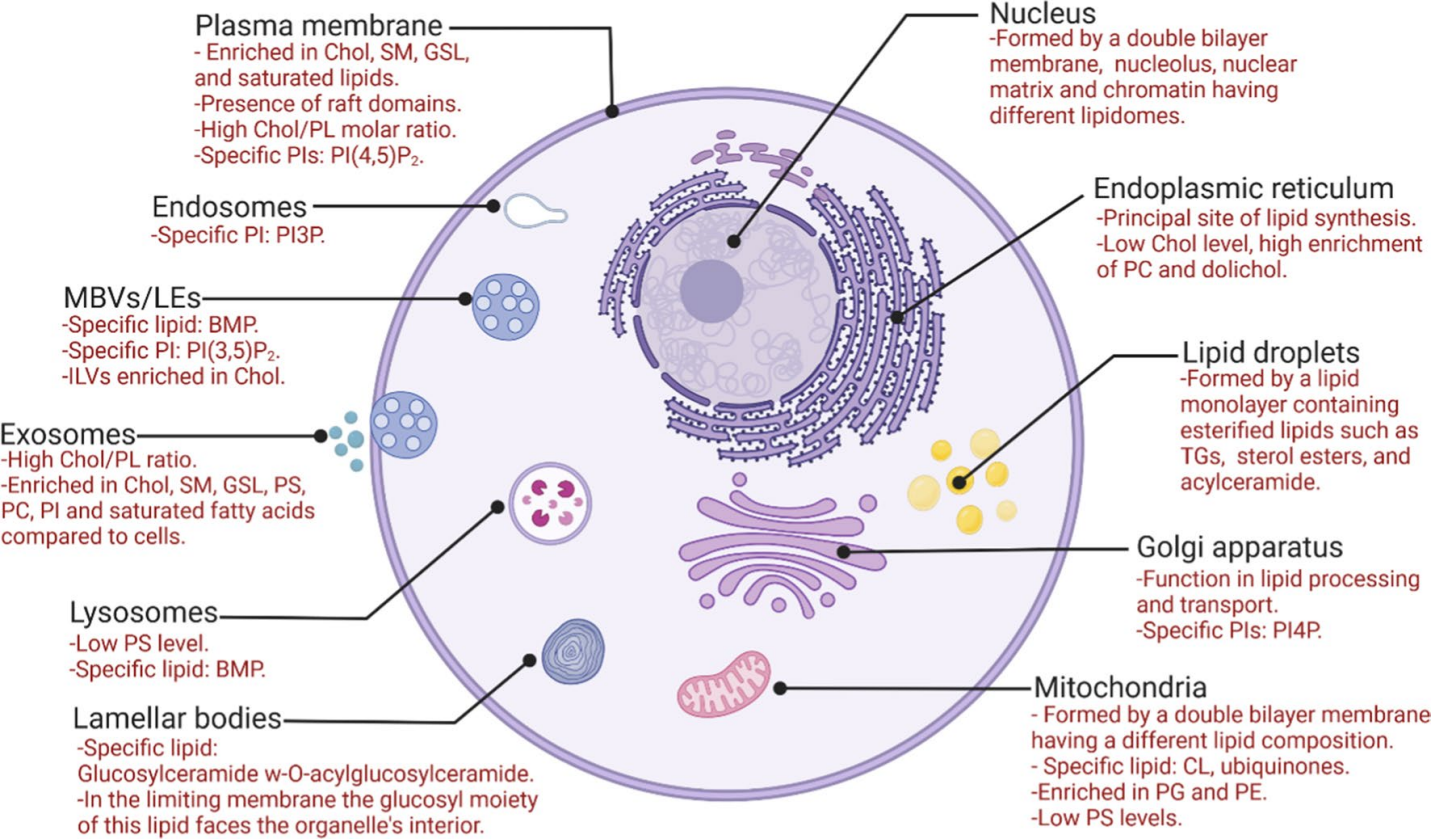
Main lipid-related characteristics of cellular compartments in mammalian cells. The figure outlines the main lipid-related characteristic of the nucleus, endoplasmic reticulum, Golgi apparatus, plasma membrane, organelles of the endosomal pathway, mitochondria, lipid droplets, lamellar bodies and exosomes. Figure created with BioRender.com. *BMP* bis(monoacylglyceryl)phosphate. *Chol*, cholesterol, *CL* cardiolipin, *GSL* glycosphingolipids, *PC* phosphatidylcholine, *PE* phosphatidylethanolamine, *PG* phosphatidylglycerol, *PI* phosphatidylinositol, *PI3P* phosphatidylinositol 3-phosphate, *PI(3,5)P2* phosphatidylinositol 3,5-bisphosphate, *PI4P* phosphatidylinositol 4-phosphate, *PI(4,5)P2* phosphatidylinositol 4,5-bisphosphate, *PL* phospholipids, *PS* phosphatidylserine, *SM* sphingomyelin, *TG* triacylglycerol. The figure is based on the information and the references included in the paper

The plasma membrane contains the highest abundance of lipids, followed by the endoplasmic reticulum, mitochondria, nuclei and microsomes, while the cytoplasm accounts for the lowest levels of lipid molecules [47, 48]. Although several similarities have been found between the lipidomes of different subcellular compartments (e.g., PC is the most abundant phospholipid in many organelles, see the sections below), the number of individual lipid species and sometimes even the abundance of lipid classes vary considerably. The subcellular organelles covered in this review along with their characteristic lipids are shown in Fig. 5.

In the sections below, we provide an overview of the subcellular fractionation protocols often used to separate cellular organelles and their lipid constituents.

### Subcellular fractionation — an essential step in mapping subcellular lipidomes

**Fig. 6:**
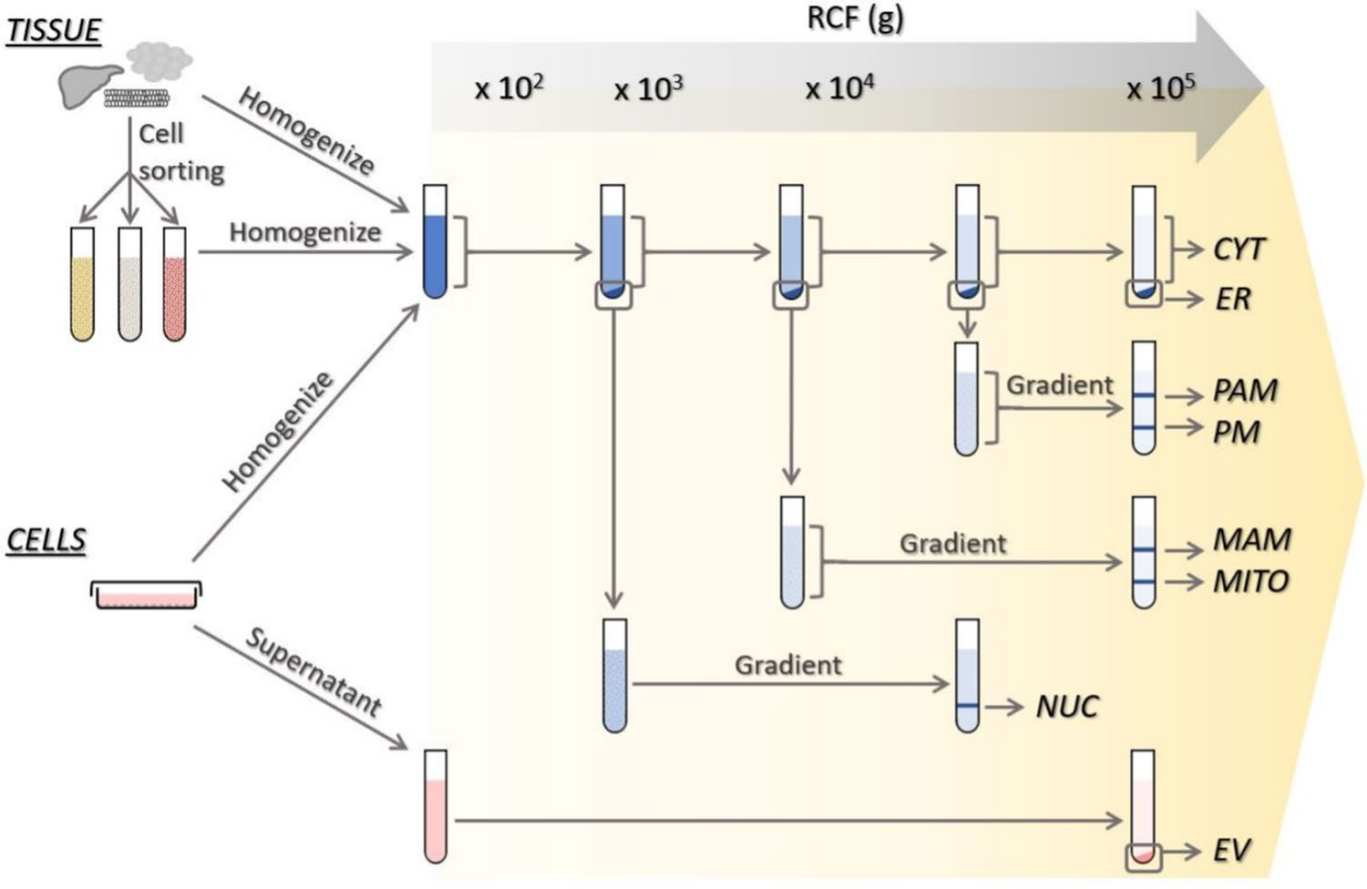
Schematic representation of a typical subcellular fractionation protocol with tissues and cell cultures. The approximate centrifugation force (g) used for fractionation of subcellular compartments via differential and density gradient centrifugation is shown at the top of the figure. For extracellular vesicles, sequential centrifugation is used. Notably, EVs can be isolated from tissue. *CYT* cytoplasm, *ER* endoplasmic reticulum, *EV* extracellular vesicle, *GA* Golgi apparatus, *MAM* mitochondrion-associated membrane, *MITO* mitochondria, *NUC* nucleus, *PAM* plasma membrane-associated membranes, *PM* plasma membrane

The tools for studying lipids include various research methods, with mass spectrometry (MS)-based lipidomics among the most popular tool used in recent years for lipid profiling [49]. These tools allow the analysis of lipid classes, their localization, determination of lipid structure and quantitation [37, 50]. This lipid analysis usually starts with subcellular fractionation (Fig. 6) and lipid extraction, followed by lipid species separation, detection, and identification.

To understand the involvement of the subcellular lipidome in cellular processes in complex samples, such as tissues, the lipids first need to be mechanically and enzymatically but gently processed to obtain single-cell suspensions. When necessary, different cell types can be separated into distinct cell fractions (e.g., by fluorescence-activated cell sorting), such that each sample is composed of a specific cell type of interest. Cell suspensions are subsequently homogenized on ice. In the classical approach, organelle separation for lipid analysis is performed via differential or density gradient centrifugation protocols similar to those used for protein-oriented research [51]. Since the nucleus is the heaviest organelle in the cell, nuclear fraction together in cell debris is usually the first fraction to be collected using low-speed centrifugation (Fig. 6) [52–54]. The specificity of the final product, however, strongly depends on the lysis protocol and/or purification of the nuclei. The type of cell lysis procedure (depending on the cells) as well as the use of Triton X-100 might influence the degree of nuclear fraction contamination with other cellular components, especially the endoplasmic reticulum. Interestingly, Triton X-100 has is also used to separate nuclear matrix lipids from the nuclear envelope pool [7]. Additional centrifugation can help eliminate cell debris that initially coprecipitates with nuclei. After nuclear separation, the application of a higher centrifugal force (e.g., 8000×*g*) produces mitochondrion- and mitochondria-associated membrane pellets, while the plasma membrane, endosomes/lysosomes, endoplasmic reticulum and Golgi apparatus are among the organelles that remain in the supernatant [48]. The plasma membrane and associated membranes are the next fraction to be pelleted when the remaining supernatant is subjected to high-speed centrifugation, e.g., 25,000×*g*. The use of gradient centrifugation is a critical step for separating mitochondria from mitochondria-associated membranes or the plasma membrane from plasma membrane-associated fractions. A comparison of different methodologies for mitochondria isolation demonstrated that the purest mitochondria samples are obtained via differential centrifugation followed by ultracentrifugation in a density gradient, while lower purities are obtained with magnetic bead-assisted isolation or differential centrifugation alone [55]. Ultracentrifugation of the final supernatant at or higher than 100,000 × *g* results in the separation of cytoplasm from the organelles remaining in the sample. Due to their low density, lipid droplets float on top of the aqueous gradient after ultracentrifugation. The lipid droplet fraction can be collected at this stage and washed via centrifugation at 20,000×g several times to remove contaminant membranes and proteins, and thus, the desired purity is reached [56]. The ultracentrifugation speed can be modified to isolate lipid droplets of specific sizes, i.e., lower speed ultracentrifugation (e.g., 10.000×*g*) is sufficient for the isolation of large lipid droplets, such as those present in the liver and white and brown adipose tissue [57, 58]. The endoplasmic reticulum fraction is usually isolated using a discontinuous sucrose gradient. By loading the supernatant onto a three-layered sucrose gradient, the endoplasmic reticulum fraction is separated by high rotational speed centrifugation, higher than 100,000×*g* [59]. To obtain the Golgi apparatus-enriched fraction, density gradient separation at ultrahigh speed is required [60–63]. The purity of the fractions is analyzed by measuring the levels of marker proteins and/or lipids known to be enriched in the organelle of interest [37].

The separation of the organelles in the endocytic pathway is challenging due to their similar sizes and/or densities. However, several strategies have been used to increase yield [64, 65]. For example, Tharkeshwar et al*.* isolated multivesicular bodies (MVBs)/lysosomes and the plasma membrane using superparamagnetic iron oxide nanoparticles, and then the lipidome of these organelles was analyzed via liquid chromatography with tandem mass spectrometry (LC‒MS/MS) [64].

Extracellular vesicles are released by cells. They are formed via two main mechanisms: direct budding from the plasma membrane (ectosomes/microvesicles) or fusion of the limiting membrane of MVBs with the plasma membrane (exosomes). In fact, exosomes correspond to the intraluminal vesicles of MVBs that fuse with the plasma membrane. These two types of extracellular vesicles are often been separated by sequential centrifugation on the basis of their different sizes (Fig. 6) [66–71]. However, their sizes have recently been shown to overlap more than previously thought, and they can be co-pelleted to some extent. Several studies have identified proteins that are enriched in specific populations of extracellular vesicles and their levels can be measured to characterize the purity of a sample [66, 72, 73]. In addition, a combination of different strategies, such as immunoaffinity purification utilizing antibodies specific for markers abundant in fractions of interest, may be employed to increase the validity of organelle lipidome study results. These processes include, for example, immunoaffinity purification of the endoplasmic reticulum [74], Golgi [75] or plasma membrane [76].

Following subcellular fractionation and lipid isolation from subcellular compartments, gas chromatography coupled to mass spectrometry (GC‒MS) or LC‒MS and shotgun lipidomic analysis are among the most popular methods used for lipid analysis in the past decade [49]. To characterize the lipid composition of cellular organelles completely, MS and other advanced methods need to be used [37]. The methods used for the lipidome profiling of subcellular compartments are given in Table 1. However, a detailed discussion of these methods is beyond the scope of this review and can be found elsewhere [49, 77–80].

**Table 1:** Lipid composition of subcellular compartments

Cell/Tissue type	Organelle	Lipids	Method of detection	References
Mouse liver	Nucleus	GP (PC, PE, PI, PS, LPE, PG, PA), SP (Cer, SM)	Direct infusion-MS	[33]
Sprague Dawley female rat liver	Nucleus (nuclear envelope, matrix, microdomains)	GP (PC), ST(Chol), SP (SM)	Inorganic phosphorus quantification; TLC	[7]
Human and mouse lumbar spinal cord	Nucleus	GL (DG, TG), GP (PC, PE, PS, PG, PA, LPA), SP (Cer, GlcCer, LacCer)	LC–MS	[89]
Bovine liver	Nucleus (nuclear envelope)	FA, GL (DG, TG), GP (PC, PE, PS, PI, LPC, CL), SP (SM), ST (Chol)	Inorganic phosphorus quantification, TLC, GC, densitometry	[90]
Rat liver	Nucleus (nuclear envelope)	GL, GP (PC, PE, PI, PS)	HPLC; TLC	[91]
Rat heart	Nucleus	GP (PC, PE, etherPC, etherPE)	ESI–MS	[92]
HEK 293T cells	Nucleus	GP (PC, LPC, PE, etherPC, PI, LPI, CL, PG, PA, PS), SP (SM)	Inorganic phosphates measurement, NMR	[237]
Cells: CHO, HCC1806, HEK, hMSC, HMLE, MDCK, RBL-2H3	Plasma membrane	GP (PC, PE, PI, PG, PS), SP (SM), ST (Chol), FA (PUFA, MUFA)	Direct infusion-MS/MS	[97]
Human platelets	Plasma membrane	GP (PC), SP (SM), ST (Chol)	LC–MS/MS, GC–MS	[99]
Red blood cells	Plasma membrane	GP (PC, PE, PS), SP (SM)	LC–MS/MS	[238]
Cells: HPAEC, HLF, HUVEC, HeLa, HEK293, MEF	Plasma membrane	ST (Chol)	Quantitative Image Analysis	[98]
Red blood cells	Plasma membrane	FA (PUFA)	GC-FID	[239]
NB100 cells	Plasma membrane	FA (PUFA, MUFA)	GC	[240]
MA-10 cells	Plasma membrane, endoplasmic reticulum, mitochondria, plasma membrane-associated membrane, MAM, cytoplasm	GP (PC, PE, PS, PI, PA, CL), SP (SM, Cer), ST (CE),	TLC, LC–MS, LC–MS/MS	[48]
Human fibroblasts	Mitochondria-ER fraction	GP (PC, PE, PS, PI, PG, CL, lysophospholipids), SP (SM, Cer, gangliosides), GL (DG, TG)	UPLC-HRMS	[241]
RAW264.7 cells	Nuclei, mitochondria, endoplasmic reticulum, plasmalemma, dense microsomes, and cytoplasm	GP (PC, PE, PI, PA, etherPC, etherPE), SP (Cer), ST (Chol, lanosterol, desmosterol, 7-dehydroChol, oxidized Chol), PR (dolichol)	LC–MS, LC–MS/MS	[47]
Human liver and skeletal muscle tissue	Mitochondria	GP (PC, PC plasmalogens, PE, PE plasmalogens, PG, PI, PS, PS plasmalogens, CL, LPC, LPE, LPI), SP (SM, Cer), GL(TG), FFA	UHPLC-MS/MS	[108]
Human liver	Mitochondria	GP (PC, PE, PS, PI, PA, CL), SP (SM), ST (Chol), GL (TG), FFA	TLC, GC	[105]
Rat liver	Mitochondria	GP (PC, PE, PG, PI, PS, CL, LPC, LPE), SP (SM, Cer, gangliosides), GL (MG, DG, TG), FA, PR	LC–MS/MS	[109]
Mouse brain	Mitochondria	GP (PC, etherPC, PE, etherPE, PG, PI, PS, CL, LPC), SP (SM, Cer), ST (Chol)	LC–MS/MS	[106]
Mongolian gerbils muscle and brown adipose tissue	Mitochondria	GP (PC, PE, PG, PI, PS, CL, LPC, LPG, LPE, LPS), SP (SM), FFA	LC–MS/MS	[242]
Mouse liver	Mitochondria	GP (PC, PE, LPC, LPE), SP (Cer, HexCer, dhCer, SM)	LC–MS/MS	[243]
Mouse liver	Mitochondria	GP (PA, PC, PE, PG, PI, PS, CL, LPC, LPE, LPI), SP (SM, Cer), GL (DG, TG), FA	UPLC-HRMS	[244]
MIN6 β-cells	Golgi apparatus, mitochondria, plasma membrane, endoplasmic reticulum, lysosomes	GP (PC/PE ratio), SP (Cer, GlcCer, SM)	LC–MS/MS	[245]
Human fibroblasts	Mitochondria-endoplasmic reticulum fraction	GP (PC, PE, PS, PI, PGs, CL, lysophospholipids), SP (SM, Cer, gangliosides), GL (DG, TG)	UPLC-HRMS	[117]
Rat liver homogenate	Golgi apparatus	GP (PS, PI, PE, PC, LPC), SM neutral lipids	TLC and fluorography	[131]
Mouse liver homogenate	Golgi apparatus	GP, Chol, CE, TG	Beta- and gamma-scintillation spectrometry	[63]
Neural retina	Golgi apparatus	SP (GM3, GD3, GT3 gangliosides)	TLC and fluorography	[133]
Fibroblasts	Golgi apparatus	SP	C5-DMB-GalCer, Confocal microscopy	[246]
Keratinocytes	Golgi apparatus, plasma membrane	SP (SM)	C6-NBD-Cer, Confocal microscopy	[247]
PC12D cells	Golgi apparatus, plasma membrane	GSL	LacCer-BODIPY, nanoLC-FLD-ESI–MS	[248]
HeLa cells	Golgi apparatus	PI4P	Immunofluorescence by confocal microscopy	[125]
HeLa cells	Golgi apparatus	SP	Confocal and STED microscopy	[249]
HeLa cells	Late endosomes/ lysosomes, plasma membrane, cell lysate	GP (including LPC, LPE, LPI, LPS, LPA, etherPC, etherPE), SP, ST (Chol), Storage lipids	LC–MS/MS	[64]
BHK cells	Late endosomes	GP (including BMP), SM	GLC-MS, TLC and autoradiography	[175]
Human B lymphocytes	Endocytic organelles	GP (including BMP), ST (Chol)	Immuno-electron microscopy	[176]
Mouse liver	Lysosomes	GP (PC, PE, PS, PI, LPC, LPE), SP (SM, Cer, HexCer), GL (DG, TG), ST (CE)	Infusion-based MS/MS	[187]
Human semen	Extracellular vesicles	GP (including LPC, LPE, LPS), SP (SM), ST (Chol)	TLC, Chol analyzed enzymatically	[196]
Guinea pig reticulocytes	Extracellular vesicles	GP	TLC	[197]
RN cells (HLA-DR15 +)	Extracellular vesicles	GP (PC, PE, etherPE, PI, PS, PA, BMP, CL), SP (SM, GM3), ST (Chol)	TLC, MS/MS, electron microscopy (Chol)	[198]
MLP29 and RH cells	Extracellular vesicles	GP, SP (SM, Cer), GL (DG, TG), ST (CE)	UHPLC-MS	[199]
LIM1215 cells	Extracellular vesicles	GP, SP, ST, GL	HCD-MS/MS	[200]
Oli-neu cells	Extracellular vesicles	GP (PC, PE, PS), SP (SM, Cer, HexCer), ST (Chol)	NanoLC-MS/MS	[201]
PC-3 cells	Extracellular vesicles	GP, SP (Cer, SM, GSL), ST (Chol, CE), GL (DG)	UHPLC-MS	[202]
Human semen	Extracellular vesicles	GP (PC, PE, PS, PI), SP (SM), ST (Chol)	TLC, GC	[203]
Human urine	Extracellular vesicles	GP, SP (SM, Cer, GSL), ST (Chol, CE), GL (DG)	UHPLC-MS	[204]
Human serum	Extracellular vesicles	GP, SP (SM, Cer, HexCer), GL (DG, TG), ST (CE)	MS/MS	[205]
Cell lines: B16-F10, MDA-MB-231–4175 and AsPC-1	Extracellular vesicles	GP (including CL), SP (SM, Cer), GL (MG, DG, TG)	LC–MS/MS	[206]
Human platelets	Extracellular vesicles	GP (including CL and BMP), SP, ST (Chol)	LC–MS/MS	[207]
Pig epidermis	Lamellar bodies	SP (Cer)	TLC	[229]
Fetal rat skin	Lamellar bodies	SP (including Cer and GlcCer)	TLC	[230]
Murine hepatocytes	Lipid droplets	GP, GL (DG, TG)	LC–MS/MS	[146]

*AsPC-1* human pancreas adenocarcinoma cell line, *B16-F10* mouse melanoma cell line, *BHK* baby hamster kidney cell, *BMP* bis(monoacylglyceryl)phosphate, *CE* cholesteryl ester, *Cer* ceramide, *CHO* Chinese hamster ovary cells, *Chol* cholesterol, *CL* cardiolipin, *DG* diacylglycerol, *dhCer* dihydroceramide, *FA* fatty acyls, *FFA* free fatty acids, *FLD* fluorescence detection, *GC* gas chromatography, *GL* glycerolipids, *GlcCer* glucosylceramide, *GLC* gas liquid chromatography, *GP* glycerophospholipids, *GSL* glycosphingolipids, *HCC1806* breast cancer cell line, *HEK293* human embryonic kidney 293 cells, *HeLa* human cervical carcinoma cells, *HexCer* hexosylceramide, *HLF* human lung fibroblasts, *HMLE* human mammary epithelial cells, *hMSC* human bone marrow derived mesenchymal stem cells, *HPAEC* human pulmonary artery endothelial cells, *HUVEC* human umbilical vein endothelial cells, *LacCer* lactosylceramide, *LIM1215* human colorectal cancer cells, *LPA* lysophosphatidic acid, *LPC* lysophosphatidylcholine, *LPE* lysophosphatidylethanolamine, *LPG* lysophosphatidylglycerol, *LPI* lysophosphatidylinositol, *LPS* lysophosphatidylserine, *MA-10* mouse steroidogenic MA-10 tumor Leydig cells, *MAM* mitochondrial associated membrane, *MDA-MB-231–4175* breast adenocarcinoma cell line, *MDCK* Madin-Darby canine kidney cells, *MEF* mouse embryonic fibroblast, *MG* monoacylglycerol, *MIN6 β* mouse insulinoma β cell line, *MLP29* mouse liver progenitor cells, *MUFA* monounsaturated fatty acids, *NB100* human primary neuroblastoma cells, *Oli-neu* mouse oligodendroglial cell line, *PA* phosphatidic acid, *PC* phosphatidylcholine, *PC-3* human prostate cancer cell line, *PC12D* pheochromocytoma of the rat adrenal medulla, *PE* phosphatidylethanolamine, *PG* phosphatidylglycerol, *PI* phosphatidylinositol, *PI4P* phosphatidylinositol 4-phosphate, *PR* prenol lipids, *PS* phosphatidylserine, *PUFA* polyunsaturated fatty acids, *RAW264.7* immortalized mouse macrophage-like cells, *RBL-2H3* basophilic leukemia cell line, *RH* primary hepatocytes, *RN* human B cells, *SM* sphingomyelin, *SP* sphingolipids, *ST* sterol lipids, *TG* triacylglycerol, *TLC* thin layer chromatography

### Lipid composition of the nucleus

In the mammalian nucleus, lipids are not only constituents of nuclear envelope membranes but are also present in the nucleolus, nuclear matrix and chromatin [7–9]. They have even been shown to organize (within the nucleoplasm) into structures called nuclear lipid microdomains [7]. In general, nuclear lipids play both structural and functional roles, depending on their specific location. They contribute to the maintenance of some of the fundamental structural features of nuclei. For example, nuclear lipids regulate the fluidity of both the nuclear envelope and nuclear matrix (PC/SM/Chol ratio) and function as anchorage points for chromatin (e.g., PS, facilitating the renucleation of the nuclear envelope after cell division) [24, 81–83]. Moreover, nuclear lipids are involved in the regulation of DNA replication, transcription and gene expression, and they also function as platforms in signal transduction pathways related to hormone and vitamin signaling [8, 24, 84, 85]. Notably, the nucleus carries some of the enzymatic machinery required for autonomous lipid metabolism [86, 87]. Several enzymes involved in sphingolipid metabolism, for example, can be found in the eukaryotic nucleus; these enzymes include sphingosine kinase, sphingomyelinase and sphingomyelin (SM) synthase, among others [24, 84, 87]. Although nuclear lipids seem to be vital for proper cell maintenance and health, studies on the mammalian nuclear lipidome are rare, and few have reported on the same lipid pools [88]. This death of information may be, at least in part, attributed to the efficiency of nucleus isolation and purification, which are essential for subsequent profiling of lipids. Unfortunately, information on the final characterization of purified nuclei is frequently missing in the reported studies, making it virtually impossible to directly compare the lipidomic results. Nevertheless, some features of the mammalian nuclear lipidome have been commonly reported in most studies [9, 33, 84, 87, 89–91]. Glycerophospholipids compose the bulk of nuclear membranes, with PC constituting 30–50% of the all nuclear lipids and PE constituting ~ 20%. Other nuclear glycerophospholipids include PI, phosphoinositides (such as phosphatidylinositol 4,5-bisphosphate, PI(4,5)P_2_) and PS, phosphatidylglycerol (PG) and lysophospholipids. SM is the main sphingolipid, but Cer, a-series gangliosides (GM_1_ and GD_1_a) and other minor sphingolipid species have also been detected (see figure Fig. 3 for a schematic representation of these structures) [7, 8, 84]. The nuclear lipidome also includes Chol (with its hydroxyl and oxygenated derivatives), dolichol and ether lipids [24, 47, 92]. Interestingly, TG seems to be more abundant in the nuclear compartment than in other organelles, possibly due to the lipid metabolism that has recently been characterized in the inner nuclear membrane and the subsequent formation of nuclear lipid droplets [86, 89, 93].

For the reasons described above, the lipidome of the mammalian cell nucleus still has not been fully described, and differences among the pools of nuclear lipids are largely uncharacterized. For example, Cascianelli et al. concluded that nuclear lipid microdomains have a distinct lipid composition compared with that of nuclear membranes, especially regarding the proportions of PC, SM and Chol, which are 1.5/0.6/1 in the latter and 1/1/1 in the former [7].

Overall, although the main mammalian nuclear lipidome has previously been described, future studies will help to clarify and uniformize existing data, identify specific lipid species and characterize different lipid pools since they play fundamental roles in maintaining nuclear architecture and function.

### Lipid composition of the plasma membrane

The plasma membrane plays an essential role as a barrier between the cytoplasm and the extracellular milieu, as well as in energy storage, maintenance, transport, signal transduction and extracellular communication [24, 94, 95]. The mammalian plasma membrane consists mainly of glycerophospholipids, including PC, PE and PS, as well as SM, ganglioside and approximately 20–50% sterol [95]. PI is present in small quantities and is involved in cell signaling [50]. Moreover, PI(4,5)P_2,_ a phosphorylated PI, is enriched at the plasma membrane compared to its level in the membranes of other organelles. Plasma membrane lipids are important cell signaling messengers and are involved in cell proliferation, survival and migration, apoptosis, inflammation, insulin activation, angiogenesis, regulation and the organization of integrins [50, 96].

Several studies have been performed to determine the lipid profile of the plasma membrane. For example, isolation and gradient centrifugation of cell organelles, including the plasma membrane, followed by LC‒MS analysis, have resulted in the identification of various lipid species, including glycerophospholipids, sphingolipids and sterols [48]. Although cell lines derived from different origins share similar lipid profiles, the lipidome of the plasma membrane varies slightly between cell types [97]. For example, human embryonic kidney cells and Madin-Darby canine kidney epithelial cells harbor greater amounts of PS in the plasma membrane, while the amount of sphingolipids is lower in the plasma membrane of rat basophilic leukemia cells compared to other cells studied to date, e.g., mesenchymal stem cells (MSCs) and immortalized human mammary epithelial (HMLE) cells [97]. Similarly, the cell membrane of HMLE cells does not contain significant amounts of PUFA-containing lipids, while the plasma membrane in MSCs carries high levels of fully saturated and PUFA-containing lipids [97]. The outer leaflet of the plasma membrane is composed mostly of PC, SM and glycolipid, while the inner leaflet contains PE and PS. Moreover, due to membrane lipid flip-flop, Chol is unevenly distributed between the two leaflets, with the outer leaflet containing more Chol in mammalian cells, that is thus packed more tightly than it is in the inner leaflet [95]. Similar findings on different distributions of Chol in the outer and inner leaflets of the plasma membrane have been reported in another study [98]. Chol in the inner leaflet is presumed to be involved in various cell functions, including signal transduction through its interaction with cytosolic proteins [98]. Furthermore, the plasma membrane is characterized by relatively ordered membrane nanodomains, so-called *lipid rafts*, in the plane of the membrane formed by interactions between certain lipids such as cholesterol, relatively saturated lipids and glycosylated lipids, that recruit other lipids and proteins and have specific functions [38, 99–101]. The cell cycle, changes in lipid metabolism over time, variations in sample preparation, and other factors influence the biophysical and lipidomic properties of the plasma membrane and are all potential causes of plasma lipidome variability. The observed cell-specific variations in the plasma membrane lipidome, although minor, highlight the need for further studies to understand the impact of these variances on cell function and encourage further research into the still-unknown functions of the plasma lipidome.

Changes in the lipid profile of plasma membranes might lead to irregular signaling that causes the development of various symptoms characteristic of certain disorders. Some examples are given later.

### Lipid composition of mitochondria

Mitochondria are essential for sustaining cellular energy production and cell metabolism. They play vital roles in the production of ATP and as a source of  building blocks for biosynthetic pathways, regulation of intracellular calcium, production and scavenging of intracellular reactive oxygen species (ROS) and regulation of apoptosis. Mitochondria are composed of two membranes that change in response to different factors, such as aging and apoptosis, thus altering normal redox metabolism and various physiological and pathological processes [102–104]. Mitochondrial lipids, especially those with high PUFA content, such as cardiolipin (CL), are prone to ROS-induced damage that affects lipid composition and mitochondrial function. The effects of different factors on lipidome remodeling are discussed later.

The mitochondrial lipidome of liver cells is predominantly composed of phospholipids, with less than 15% comprising free fatty acids (FFAs), acyl glycerols and Chol [105]. Interestingly, brain mitochondria carry a much higher amount of Chol, which comprises more than 20% of the total lipid content [106]. Mitochondria have a higher abundance of PGs and ether-linked phospholipids than other subcellular compartments and contain majority of the cellular ubiquinones [47]. CL is primarily present in mitochondria and mitochondria-associated membranes [48]. In addition, the most abundant phospholipid species reported in macrophage mitochondria are PC(36:2), PC(36:1), PC(38:0), PE(38:4), PE(36:1), PE(38:3), and PG(36:2) [47]. Moreover, some lipids, such as phosphatidic acid (PA) and PI, are present at higher amounts in mitochondria-associated membranes compared to mitochondria themselves, a distribution that is opposite that of PE [48].

A previous study identified ceramide glucosyltransferase, glucosylceramide galactosyltransferase and sialyltransferase in mitochondria-associated membranes and showed that these enzymes were instrumental in in the synthesis of gangliosides GM_3_, GD_3_ and GM_1_ (Fig. 3). This finding suggests that at least a portion of glycosphingolipids in mitochondria originate from mitochondria-associated membranes [107].

The levels of lipids in different classes in the mitochondrial lipidome vary depending on the tissue of origin. Hence, CL, lysophosphatidylethanolamine (LPE) and PG are present at higher abundance in skeletal mitochondria than in the liver [108]. CLs carrying 18:2 acyl chains are the most abundant CLs in both skeletal muscle and liver mitochondria, although their presence is higher in skeletal muscle, with CL(18:2)_4_ being the most abundant. Moreover, among the CLs present in skeletal muscle mitochondria, those containing 20:0 and 22:6 acyl chains constitute the largest group, while the level of CLs with 18:1, 20:4 and 22:2 acyl chains are higher in liver mitochondria [108]. Interestingly, the same study reported PG with 22:6 acyl chains are almost exclusively found in liver mitochondria. Moreover, although the absolute levels of the main phospholipid classes in mitochondria (PC and PE) appear to be the same among organs, PC and PE plasmalogens are present in higher amounts in skeletal muscle mitochondria than in the liver [108].

A detailed qualitative analysis of fatty acyl levels in rat liver mitochondria led to the identification of only 7 types of FFAs belonging to branched fatty acid chains, namely, acylcarnitines, octadecanoids and acylamide species. The same study reported a large variety of glycerophospholipids, sphingolipids and glycerolipids in the mitochondrial lipidome and only 3 different prenols [109].

### Lipid composition of the endoplasmic reticulum

The endoplasmic reticulum is a continuous and dynamic membrane system that is considered to be a major site for the production and modification of many proteins and lipids [4, 110]. Most membrane proteins are targeted and translocated to the endoplasmic reticulum, which acts as the point of entry to other endomembrane compartments [111]. The endoplasmic reticulum is crucial to the biosynthesis of structural phospholipids and Chol. The most abundant membrane lipids, PC and PE, as well as the least abundant lipid, PI, are produced in the endoplasmic reticulum [112]. These phospholipids are also the main lipid components of the endoplasmic reticulum, with PC being the most abundant (approximately 54%), followed by PE (approximately 20%) and PI (11%) [24]. Although Chol is produced in the endoplasmic reticulum, the abundance of Chol in this organelle is less than 8% because it is transferred to the plasma membrane immediately after synthesis [24]. Additionally, the endoplasmic reticulum carries minor lipids, including diacylglycerol (DG), cytidine diphospho-DG, PA, lysophospholipid, and dolichol [4, 25]. The reticulum plays an important role in the production of nonstructural lipids, such as TGs and cholesteryl esters [113]. Cer, the hydrophobic backbone of sphingolipids, originates from the endoplasmic reticulum [114] along with galactosylceramide [115], the major constituent of myelin. The lipids that are synthetized in the endoplasmic reticulum are distributed throughout the cell via secretory pathways and/or specialized domains, such as organelle contact sites. TG is transferred to lipid droplets that are formed on the reticulum membrane and subsequently associate with other organelles through membrane contact sites [112]. Membrane contact sites between the endoplasmic reticulum and other organelles, including the Golgi complex, mitochondria and the plasma membrane, have been recently observed [116]. These findings have led to the conclusion that the endoplasmic reticulum is physically and functionally connected to other organelles, complicating the study of separate subcellular fractions.

Venugopal et al*.* investigated subcellular lipid localization during steroidogenesis using steroidogenic MA-10 mouse tumor Leydig cells [48]. The authors aimed to determine the reorganization of membrane lipids at the subcellular level during steroidogenesis. They analyzed lipid species in organelles, including plasma membrane-associated membranes (plasma membrane, endoplasmic reticulum, and mitochondria) and mitochondrion-associated membrane (endoplasmic reticulum and mitochondria) microdomains. Venugopal et al*.* measured and compared 221 lipid species in cells treated with dibutyryl cyclic adenosine monophosphate with or without the steroidogenesis inhibitor cycloheximide and untreated control cells. They measured glycerophospholipid (PC, PE, PS, PI, PA, and CL), sphingolipid (SM, Cer), and neutral lipid cholesteryl ester levels to elucidate the roles of these lipids in steroidogenesis and membrane reorganization. They found that the endoplasmic reticulum exhibited increased levels of PA after hormonal stimulation, and this effect was attenuated by cycloheximide treatment. The authors also reported a significant decrease in PE and PS content in the endoplasmic reticulum, but the levels of these lipids were increased in the plasma membrane-associated membranes following hormonal stimulation, suggesting lateral movement of these lipid species. The data suggest that plasma membrane-associated membranes and mitochondrion-associated membrane microdomains are crucial in the process of lipid trafficking and that these membranes undergo dynamic reorganization during hormone-induced steroidogenesis.

Veyrat–Durebex and colleagues combined metabolomic and lipidomic approaches to identify metabolites and lipids involved in endoplasmic reticulum-mitochondrion metabolism [117]. They detected lipids belonging to 12 different lipid classes: PC, PE, PG, PI, PS, lysophospholipid, SM, CL, Cer, DG, ganglioside, and TG. PC and PE accounted for 60% of the total concentration of detected lipids and were present in both the endoplasmic reticulum and mitochondria. Their abundance was followed by that of SM, TG and Cer.

### Lipid composition of the Golgi apparatus

The Golgi apparatus is a cellular organelle that plays a key role in processing lipid cargo synthesized in the endoplasmic reticulum and its subsequent sorting in vesicles at the trans-Golgi network [118]. The Golgi apparatus is composed of aligned stacks of cisternae that are highly polarized, enabling cargo to be received at the cis-Golgi site and guided to the trans-Golgi, where it is exported. The distribution of enzymes, resident proteins and lipid composition in the Golgi apparatus differs throughout, from the cis-Golgi to the trans-Golgi. Similarly, the luminal pH, is gradually changed, from pH 6.7–6.0, which is essential for cargo processing [118, 119].

Glycosphingolipids [120–122], phosphatidylinositol 4-phosphate (PI4P) [123] and DGs [124] are primary and secondary messengers related to signaling involving the Golgi. PI4P turnover at the Golgi complex is regulated by sphingolipid metabolic flux, revealing a two-way relationship between sphingolipids and phosphoinositide metabolism in this organelle [125]. In vitro studies based on HeLa cells and by confocal microscopy revealed the colocalization of the cis-Golgi protein marker G95 and a PI4P marker.

Cer is produced de novo in the endoplasmic reticulum and, when transported to cis-Golgi, may be converted to glucosylceramide. PI4P mediates the transfer of glucosylceramide to the trans-Golgi network, where it is converted to SM or glycosphingolipid [126, 127]. Glycosphingolipids are synthesized de novo in the Golgi apparatus and in the endoplasmic reticulum and then transported to the outer leaflet of the plasma membrane, where they play a key role in numerous signaling processes [128]. Glycosylation in the Golgi apparatus is essential for glycosphingolipid biosynthesis. The synthesis of glucosylceramide takes place on the cytosolic surface of the Golgi, and a floppase must be transported to the Golgi lumen [126] to convert glucosylceramide into a glycosphingolipid. The synthesis of glucosylceramide in the Golgi apparatus has been shown to be a process independent of the Cer transfer protein [127]. Other lipids along with different sensors and effectors are transported within the Golgi apparatus to trans-Golgi membranes [50]. The lipid composition of the Golgi inner membrane differs from that of the trans-Golgi network. For example, in the trans-Golgi network, some of the Golgi PC is replaced by newly synthetized glycosphingolipids, accompanied by an enrichment in Chol due to the preferential interaction of this lipid with sphingolipids [129, 130], facilitating Golgi vesicle budding [50]. After separating the membrane and the interior content fractions of the Golgi, Howell and Palade concluded that PC is the most abundant phospholipid in both the membrane and interior of the Golgi, while PS and PE are more abundant in the membrane fraction [131]. In contrast, the majority of Golgi TGs is found in the content fraction. Moreover, Chol is heterogeneously distributed throughout the Golgi apparatus, with the highest amount at the trans-Golgi [132]. Furthermore, the GM3, GD3 and GT3, precursors of more complex gangliosides of the a-, b- and c-series, are synthesized in the proximal Golgi, although in different compartments. GM_3_ and GD_3_ have been identified in the cis/medial Golgi, while the specific synthesis of GT_3_ is located in the trans Golgi compartment [133].

### Lipid composition of lipid droplets

Lipid droplets are structurally unique among organelles with a dense hydrophobic core comprising neutral lipids and enveloped with a phospholipid monolayer [134]. Although they have long been regarded as passive energy repositories, in the last decade, the dynamic functions of lipid droplets have been revealed, as they have been found to be immersed in all aspects of cellular function [1, 135]. Lipid droplets are formed in the endoplasmic reticulum, where the size of a nascent neutral lipid lens composed of newly synthesized TGs and sterol esters increases between the two leaflets of the endoplasmic reticulum membrane [136]. These nascent lipid droplets bud from the reticulum membrane and are released into the cytosol, where their size further increases or decreases and where they contact other organelles [137–140].

One of the essential biological functions of lipid droplets involves their dynamic responses to changes in cellular nutrient requirements, which fluctuate, and environmental-induced signaling to coordinate lipid metabolism and thus meet the energy demands for membrane biosynthesis, cell growth and survival [138, 141]. Lipid droplets are continuously formed and broken down in a cell, functioning simultaneously as sinks that protect membranes and organelles from lipid overload and as sources of lipids for essential processes, such as those needed for balancing membrane saturation levels and fatty acids needed for mitochondrial energy production [142–144]. In stressed cells, lipid droplet size, subcellular location, and lipid and protein composition can rapidly change [145]. For example, low calorie intake has been reported to result in the enrichment of hepatocyte lipid droplets with TGs containing long-chain PUFAs, while hepatocyte lipid droplets from mice fed a high-fat diet contained fewer unsaturated TGs [146]. Lipid droplets provide lipids that may directly function as signaling molecules, such as fatty acids, or as biosynthetic precursors for other bioactive lipid mediators, such as eicosanoids, retinoic acid, endocannabinoids, and Cer [147–149]. Remarkably, lipid droplets sequester and release various proteins, thereby affecting protein turnover, signaling pathways and gene transcription [45, 150]. Lipid droplets may store lipophilic drugs and control drug efficacy by altering their subcellular distribution [151].

The neutral lipid droplet core primarily stores lipids in their esterified storage forms; e.g., fatty acids are stored as TGs, sterols are stored in the form of sterol esters, retinoic acid is stored as a retinyl ester, and Cer is stored as an acyl-Cer [147, 152, 153]. Lipid droplets may also harbor varying amounts of lipid intermediates produced during neutral lipid biosynthesis and breakdown, most notably DG but also lysophosphatidic acid and PA. Lipid droplets can also store significant amounts of ether lipids [154]. In alignment with tissue-specific functions and storage requirements, lipid droplets in different cells and tissues may exhibit significant differences in the relative proportions of these major lipid species. Lipid droplets in adipocytes are predominantly composed of TGs, whereas lipid droplets in steroidogenic cells or macrophage foam cells carry mostly cholesteryl esters [155–157]. On the other hand, hepatic stellate cells, which are specialized in the storage of retinol (vitamin A), harbor LDs that are enriched with retinyl esters [158, 159]. The surface monolayer of mammalian cell lipid droplets includes electroneutral phospholipid species as well as some sterols. It is primarily composed of PC, followed by PE and PI, and significant amounts of corresponding lysophospholipid species may also be found [154].

Lipid droplets play major roles as regulators of fatty acid trafficking, metabolism and signaling. Consistent with these functions, the fatty acyl chain composition of neutral lipids stored within lipid droplets differs between cell types and is dynamically altered during cell state transitions. For instance, channeling of SFAs into TGs may reduce TG availability for conversion into Cer, thus limiting both palmitate- and Cer-induced lipotoxicity and inflammatory signaling [160–162]. In addition, the esterification and release of Cer from lipid droplets modulates the activation of Cer-induced signaling pathways in a cell [153]. Lipid droplets in hypoxic kidney cancer cells release unsaturated fatty acids that replace saturated acyl chains in cell membranes, preventing endoplasmic reticulum stress [142].

Lipid droplets may function as sinks for PUFAs, which limit the availability of PUFAs needed as pro-inflammatory lipid mediators, preventing inflammation [163–166]. However, when cells undergo PUFA overload that exceeds the storage capacity of lipid droplets, the lipolytic release of PUFAs from lipid droplets induces oxidative stress and cell damage, which can lead to ferroptotic cell death [166–169]. Finally, the presence of oxidized lipids in lipid droplets may alter their function and disrupt other cellular processes [164–166]. For example, oxidized and truncated TGs alter dendritic cell function by covalently immobilizing the chaperone proteins required for antigen presentation to the lipid droplet surface [45]. Additionally, peroxidized lipids have been found in lipid droplets of *Drosophila* glial cells, and they have been associated with mitochondrial dysfunction and neurodegeneration [170]. Lipid droplets are thus emerging as active regulators of lipid trafficking, which affects a wide range of cellular processes, but much remains to be learned about the lipid droplet lipidome, its dynamic changes and functions.

### Lipid composition of the organelles in the endosomal pathway

Through the endocytic pathway, extracellular material is taken up by cells, sorted and transported via vesicles to their destination [171]. Endocytosis takes place at the plasma membrane, the lipid composition of which was discussed above. In addition, the endocytic pathway involves three main organelles: endosomes, late endosomes/MVBs and lysosomes. The luminal pH of these organelles becomes gradually increasingly acidic, reaching a value of 4.5–5 in lysosomes [172]. Several differences in the lipid composition of these organelles have been identified [173, 174], including distinct membrane components of MVBs [175, 176], which include the MVB limiting membrane and intraluminal vesicles (ILVs) formed by invaginations of this membrane.

The amount of Chol varies in the organelles of the endocytic pathway. The plasma membrane contains the highest Chol-to-phospholipid ratio, which is approximately equal to one, in cells [4]. Using immunoelectron microscopy, Möbius et al. found that MVBs, particularly ILVs, contained high amounts of Chol, with lower levels in recycling endosomes and lysosomes, where Chol is largely absent [176].

Phospholipids constitute a large fraction of the total lipids in cellular membranes. Leventis and Grinstein summarized the percentage of the phospholipid classes PC, PE, PI and PS in terms of their percentage in the total phospholipid composition in different cellular compartments, including the plasma membrane, early endosomes and late endosomes [177]. PC was the most abundant phospholipid (42–49%), followed by PE (18–25%). The most remarkable difference between these compartments was the level of PS, which was 12% in the plasma membrane, 8.5% in early endosomes and 2.5–3.9% in late endosomes. In addition, higher amounts of PI were found in early and late endosomes than were found in the plasma membrane. The phospholipid composition of rat liver lysosomes and the plasma membrane has also been summarized [178]. In this study, PC was also shown to be the most abundant phospholipid (~ 40% of total phospholipids), followed by PE (~ 20% of total phospholipids). The main difference between these compartments was that the SM and PS percentages were higher in the plasma membrane [178]. However, as mentioned by the authors of these papers, these results are based on subcellular fractionation (Fig. 6), which can lead to data distortion caused by the possible isolation of impure fractions and disruption of lipids during assay procedures [177, 178].

Some phosphoinositides, which are derivatives of PI that are phosphorylated at one, two or three positions of the inositol ring, are enriched in the organelles of the endocytic pathway due to the functions of specific lipid kinases and phosphatases. Therefore, these lipids are often used to identify these compartments; for example, PI(4,5)P_2_ and phosphatidylinositol (3,4,5)-trisphosphate (PI(3,4,5)P_3_) are often enriched on the plasma membrane, phosphatidylinositol 3-phosphate (PI3P) is enriched on the membranes of early endosomes, and phosphatidylinositol 3,5-bisphosphate (PI(3,5)P_2_) is enriched in MVBs [179–181]. Moreover, phosphoinositides are involved in different cellular processes involving these organelles by recruiting proteins with specific binding domains to the cellular compartment where these lipids are located; for example, PI3P recruits proteins containing FYVE and PX domains to endosomes [182, 183].

Interestingly, in the cells of mammals and other higher eukaryotes, MBVs and lysosomes contain an atypical anionic phospholipid called bis(monoacylglyceryl)phosphate (BMP), also known as lysobisphosphatidic acid. This lipid is found in large amounts in these organelles (15–20 mol% of all phospholipids), mainly in ILVs, but are absent at other cellular locations [184–186]. In fact, ILVs may consist of at least two different populations, with one population with PC and the with BMP as the main phospholipid [175]. A main function of BMP lipid is control of the endosomal levels of Chol and sphingolipids, and it has also been shown to play a role, together with the Alix protein, in the formation of ILVs [185, 186].

Overall, we have acquired an understanding of the lipid classes and how they differ in the organelles of the endocytic pathway, but few studies have sought to identify the specific lipid species that comprise these organelles. As previously mentioned, these organelles are difficult to separate, which makes the preparation of pure samples for lipidomic analysis difficult. However, some strategies have been used to increase the purity of samples [64, 65]. For example, Tharkeshwar et al. isolated MVBs/lysosomes and plasma membranes using superparamagnetic iron oxide nanoparticles. Then, the lipid composition of these organelles in HeLa wild-type and Niemann-Pick type C1 (NPC1)-deficient cells was analyzed by LC‒MS/MS [64]. In this study, 17 lipid classes and 551 lipid species were quantified. MBVs/lysosomes were found to be composed of approximately 60% glycerophospholipids (PC, PE, PI, PS, PG, ether-linked PC/PE (PC O-/PE O-), PA and DG; 4.6% sphingolipids (Cer, hexosylceramide (HexCer) and SM); 7.5% storage lipids (TGs and Chol esters); and 28% Chol. The proportion of these lipids in the plasma membrane were approximately 62%, 4%, 0.5% and 32%, respectively. Differences in the amounts of individual lipid species between these two compartments were not specifically analyzed, but several differences were found in the lipid species of MVBs/lysosomes when control cells and NPC1-deficient cells were compared. In addition, an MS/MS lipidomic analysis of lysosome-enriched liver fractions in wild-type and granulin mutant mice was performed and the lipidomes were compared [187]. Approximately 500 lipid species were identified, and the proportions of TG, PS, PE and DG were compared, but the lysosomal lipid composition of the lysosomal fractions from the wild-type mouse liver was not individually analyzed.

In conclusion, there is still much to learn about the lipid species in the organelles of the endocytic pathway, and hopefully, future lipidomic studies will reveal the full lipid composition of these organelles.

### Lipid composition of extracellular vesicles

Extracellular vesicles are vesicles that are released by cells into the extracellular environment [188–191]. Living cells release two main populations of vesicles known as exosomes and microvesicles, which are named based on the mechanism underlying their release: exosomes correspond to the ILVs of the MVBs that fuse with the plasma membrane, and microvesicles bud directly from the plasma membrane. The sizes of exosomes and microvesicles overlap to some extent, with exosomes in the smaller range (~ 30–150 nm in diameter; ILVs have a mean diameter of approximately 50 nm in mammalian cells [192]) compared to that of microvesicles (~ 50–1000 nm in diameter) [193, 194]. It can then be expected that the lipid composition of exosomes and microvesicles resembles the composition of ILVs and the plasma membrane, respectively.

Lipids are essential molecular components of extracellular vesicles. They affect membrane physical properties [17, 25] and may play roles in maintaining the stability of vesicles in the extracellular environment. The lipid composition of extracellular vesicles has been a subject of interest for many years. In addition to the abovementioned challenges in the separation of different extracellular vesicles populations, little is known about how different preanalytical variables affect the lipid composition of extracellular vesicles in biofluids, and some of these factors may be relevant to their effective isolation [195]. Most of the lipidomics studies have so far focused on extracellular vesicles pelleted by 100,000×*g* centrifugation. This fraction is expected to contain mainly exosomes and will be referred here as such.

The lipid composition of exosomes in seminal fluid and released by reticulocytes was investigated in the late 1980s [196, 197]. Extracellular vesicles in human seminal fluid (prostasomes) exhibited a very high Chol/phospholipid ratio [196]. Similarly, exosomes released by B-lymphocytes were enriched in Chol and sphingolipids [198]. Over the years, several MS lipidomic studies have reported the identity of several hundred lipid species in more than 20 lipid classes [199–202]. Regardless of the method used for lipid analysis, several studies have shown the enrichment of Chol, SM, glycosphingolipid (GSL), PS, PC and PI in exosomes from cells also enriched with these lipids; in contrast, the content of PE was found to be similar in the cells and exosomes isolated from their supernatants [70]. It has also been shown that exosomes contain relatively more saturated and less monounsaturated fatty acids than the cells from which they are released [201, 202]. Exosomes from seminal fluid exhibit a high degree of fatty acid saturation [203]. Recently, lipidomic analyses of extracellular vesicles in blood and urine have been performed [204, 205], which has supported the use of lipid species in extracellular vesicles as noninvasive biomarkers for identifying several diseases. The lipid composition of urinary extracellular vesicles pelleted at 100,000×*g* indicated a very high level of Chol; all the PE species identified were PE ethers; and PS 18:0/18:1 were the lipid species found at high levels after Chol [204].

Only a few studies have compared the lipid composition of different extracellular vesicle populations released from the same cell line [206, 207]. The lipid composition of several populations of extracellular vesicles released by platelets was found to vary, with the exosome-enriched fraction containing the highest content of Chol and SM [207]. The enrichment of these lipids in exosomes suggests that they have a higher lipid order than other extracellular vesicles populations, a supposition that has been supported by several studies [208, 209].

As mentioned in the previous section, the anionic phospholipid BMP is abundant in MVBs/late endosomes and are found in ILVs [175, 176, 185]. Therefore, whether this lipid is found in exosomes has been a research topic. If this is the case, then BMP can be used as a marker to differentiate exosomes from vesicles formed at the plasma membrane. Some studies have reported that BMP is not present in exosomes [176, 198, 210]. However, a prior study identified this lipid in vesicles attached to follicular dendritic cells [211]. In addition, several species of BMP have recently been identified by MS in vesicles released by cultured cortical neurons (although the absolute levels of BMP were not reported [212]), in urinary extracellular vesicles and in vesicles released by HEK293 kidney cells [213]. Furthermore, phosphoinositides, known to be enriched in the plasma membrane and in MVBs, also show the potential to be useful to differentiate exosomes from microvesicles. Interestingly, methods are currently available to detect the presence of different phosphoinositides in extracellular vesicles [214].

In conclusion, lipidome analyses of extracellular vesicleshave provided useful information to understand the lipid composition of ILVs and the plasma membrane.

### Lipid composition of epidermal lamellar bodies

Epidermal lamellar bodies are secretory organelles belonging to a class of lysosome-related organelles that are exclusive to epithelial cells, including alveolar cells in the lungs and skin and oral keratinocytes. They play important physiological functions, such as breathing and epidermal barrier formation. Here, we focus on the lamellar bodies in the skin.

The epidermal barriers is composed of the outermost layer of the skin epidermis—the stratum corneum [215]. Keratinocytes gradually differentiate and form stratified layers, simultaneously producing proteins and lipids. While the keratinocyte proteins polymerize inside the cells, forming an envelope, the lipids are secreted through lamellar bodies to create a hydrophobic barrier comprising protein-lipid composites [216]. The lipid composition of the stratum corneum consists mainly of equimolar concentrations of Cer, Chol, and FFA, with the lipid mass consisting of approximately 50% Cer, 25% Chol, 15% FFA and a small percentage of phospholipids [217–219].

Lamellar bodies are unique membrane-bound organelles composed of one or several tightly packed bilayer membranes (reviewed in [220]). They are round or oval with a diameter of approximately 200–300 nm and originate by budding off from the trans-Golgi tubuloreticular membrane system of the outermost epidermal granular cells [221, 222].

Several groups have isolated lamellar bodies-enriched fractions based on their low buoyancy, low density, or small size [223, 224]. Based on these studies, it was shown that lamellar bodies contain, in addition to lipids, enzymes involved in lipid metabolism, chymotryptic enzymes (kallikreins), protease inhibitors, cathepsins, and antimicrobial peptides, which are all essential for the formation of various skin barriers [225–228].

A direct lipid analysis of lamellar bodies-enriched fractions from rodent and pig epidermis showed that lamellar bodies contain Chol, phospholipid, glucosylceramide, and SM [229, 230]. The glucosylceramides in lamellar bodies are structurally heterogeneous, the most unique of which is linoleate-rich w–O-acyl-glucosylceramide, which has been implicated in the assembly of lamellar bodies [231]. This w–O-acyl-glucosylceramide consists of 28- to 34-carbon w-hydroxy acids with amide links to sphingosine and dihydrosphingosine bases, with glucose beta-glycosidic attachments to a primary hydroxyl group of a long chain base and linoleic acid ester links to the w-hydroxyl group. The w–O-acyl-glucosylceramide contains 74–75% ester-linked linoleate, making it a major linoleic acid carrier in viable epidermis, the layer of the skin underlying the stratum corneum. Approximately 80% of w–O-acyl-glucosylceramide has been estimated to be associated with the limiting membrane of lamellar bodies, while approximately 20% has been found in internal lamellae [232].

In addition to its composition, the lamellar body membrane, another feature distinguishes lamellar bodies from other organelles. In most biological membranes (e.g., plasma membrane and membranes of biological vesicles), glycolipids are synthesized with glucose attached to the outer surface, but in the lamellar bodies, the glucosyl moiety of acyl-glucosyl Cers in the limiting membrane is located on the inner side of the membrane [220].

At the boundary between the uppermost granular cells and the stratum corneum, lamellar bodies fuse with the plasma membrane, and the contents of the lamellar bodies are released into the intercellular space.

While enzymatically modified w–O-acyl-Cers in the limiting membrane of lamellar bodies replace the lipids in the plasma membrane, forming a cornified lipid envelope, and the extruded contents are metabolized in the intercellular space by enzymes that are cosecreted from lamellar bodies. At this time, beta-glucocerebrosidase converts glucosylceramides into Cer, acidic sphingomyelinase converts them into sphingomyelin, and phospholipases convert phospholipids into FFAs and glycerols [233].

Although the formation, structure, and secretion of lamellar bodies in epidermal keratinocytes have been the most extensively studied, lamellar bodies have also been reported in other epithelial cells (reviewed in [234]). In the keratinized oral epithelia, gingiva, and hard palate, lipids are packaged in lamellar bodies similar to those in the skin epidermis; however, the volume and density of the lamellar bodies in these tissues are lower than those in the epidermis. The Cer profile of the oral stratum corneum is similar to that of the skin stratum corneum, with the exception that although linoleate is the major (70–80%) ester-linked fatty acid in epidermal Cer, in palatal Cer, linoleate constitutes only 8.8% of the ester-linked fatty acids [235]. Because the oral stratum corneum contains fewer lipids than the epidermal stratum corneum and because these lipids are organized more loosely, the permeability of the oral epithelium is higher than that of the skin [236].

In summary, the lamellar bodies deliver lipids and other components that are essential for the formation of various epidermal barriers.

## Lipidome dynamics under physiological and pathological conditions

The cellular lipidome is highly dynamic, and the lipid composition changes in response to various factors under both physiological and pathological conditions. Altered lipid metabolism, ongoing inflammation, and oxidative stress are among the conditions that can cause cellular lipidome alterations. Moreover, subcellular lipidome dynamics have been associated with both physiological (aging) and pathological conditions, leading to a wide spectrum of neurological disorders with different etiologies. To date, changes in the lipid profile of organelles have been reported in many disorders, including cancer, metabolic and inflammatory diseases, aging, and neurodegenerative disorders. In this section, we provide a few examples illustrating the lipidome alterations in health and disease contexts. Understanding lipidome remodeling under both physiological and pathological conditions might aid in finding biomarkers and identifying potential therapeutic targets for diverse pathological conditions.

### Lipidome dynamics under physiological conditions

The organelle lipidome commonly changes during various physiological processes. Herein, examples of lipidome dynamics in steroidogenesis, thermoregulation, and inflammatory cells are presented.

During steroidogenesis induced in vitro, the lipidome undergoes dynamic remodeling. Although the total PC abundance remains unchanged, the levels of specific PCs differ. For example, the levels of PC(28:0) and PC(32:1) increase, while the level of PC(34:0) profoundly decreases during steroidogenesis in mitochondria, mitochondrion-associated membranes and plasma membrane-associated membranes, and the opposite changes have been reported for the aforementioned PC pieces in plasma membranes [48]. In contrast, steroidogenesis induced in vitro leads to elevated the levels of CL and Cer in mitochondria and the plasma membrane, as well as in their respective associated membranes, and the PA level is elevated in the endoplasmic reticulum [48].

A study on Mongolian gerbils pointed to suggested an important role for mitochondrial membrane lipidome remodeling with respect to mitochondrial respiration during acute thermoregulation [242]. The brown adipose tissue mitochondrial membrane phospholipidome was found to be prone to changes during acute thermoregulation. The most significant changes were recorded for PS(30:0), PS(40:2), and lysophosphatidylglycerol LPG(16:0) in brown adipose tissue mitochondria and for LPE(24:1) and PC(44:4) in muscle mitochondria [242]. In addition, the amount of *n*-6 FFA was higher in mitochondrial membranes at high temperatures, which may be attributable to an increase in a specific species, FFA(18:2). In contrast, the levels of FFA(21:5) and FFA(22:5, *n*-3) were reduced at high temperature [242].

Finally, lipidome perturbations in subcellular compartments is induced after inflammatory cell activation, such as after the activation of macrophages induced by lipopolysaccharide and mediated via Toll-like receptor 4 [47]. The levels of Chol precursors and Cer were increased in all fractions of macrophage-like RAW264.7 cells. However, unsaturated ether-linked PE decreased only in the endoplasmic reticulum and could be associated with the release of arachidonic acid from the endoplasmic reticulum after cell activation [47].

### Lipidome dynamics during aging and neuropathology development

Lipids are highly susceptible to excessive ROS, which may cause oxidative damage. Lipid peroxidation and its implication in physiology [104, 250] and the development and progression of major stress-associated diseases have been well documented [251, 252]. Among lipids, plasmalogens, a class of glycerophospholipids (Fig. 2), terminate lipid peroxidation and thus are sometimes termed endogenous antioxidants [253]. The development of sensitive methods for performing lipidome analysis has led to the identification of associated changes with plasmalogens under pathological conditions. The mitochondrion-endoplasmic reticulum lipidome as well as the whole-cell lipidome of amyotrophic lateral sclerosis fibroblasts exhibit changes in the level of docosahexaenoic acid 22:6(*n*-3) in phospholipids. Moreover, plasmalogens containing FFA(22:6) are major phospholipids useful for discriminating between amyotrophic lateral sclerosis and control samples [241]. Additionally, amyotrophic lateral sclerosis fibroblasts exhibit changes in the sphingolipid composition of the mitochondrion-endoplasmic reticulum lipidome [241].

Aging and Parkin loss mediate brain mitochondrial lipidome remodeling, which affects several cellular functions [243]. Aging is accompanied by increases in the levels of hydroxylated hexosylceramide (OH-HexCer) and PE and a reduction in PC and Cer in mitochondrial membranes. The age-induced increase in hydroxylated Cer (OH-Cer), HexCer and CL levels and the decrease in LPE, PI, PS and lysophosphatidylcholine (LPC) levels in mitochondrial membranes are specific to parkin loss [243]. The levels of specific lipids in mitochondria are significantly reduced during aging, namely, Cer(74:8), HexCer(40:1), HexCer(42:1), and HexCer(44:2), while the levels of Cer(72:6), OH-HexCer(41:1), OH-HexCer(42:2), OH-HexCer(43:2) are increased; these changes may be attributable, at least in part, by aging-related oxidative stress [243].

Alterations in the plasma membrane lipidome have been observed in patients with autism spectrum disorder [254]. A study showed that certain processes, including the response to oxidative stress, influence PS level in the plasma membrane, causing morphological alterations in red blood cells. Decreased levels of PUFAs have been observed in children with autism spectrum disorder compared with children with normal development [239, 254, 255]. Notably, glutamate excitotoxicity in autism spectrum disorder might be associated with changes in lipid rafts in the plasma membrane, particularly alterations in the sphingolipid and Chol composition [254].

A study published by Bolognesi and colleagues identified several phospholipid fatty acids in human neuroblastoma cell membranes and reported alterations to their levels after palmitic acid supplementation [240]. Hyperactivation and alterations in several signaling pathways leads to changes in the plasma membrane lipidome of in cancer cells; for example, the desaturation rate of palmitic to palmitoleic acid by stearoyl-CoA desaturase is increased. These lipid composition alterations imply a potential association between cancer and membrane biosynthesis [256]. MIN6 β-cells exposed to palmitate exhibited a small increase in the levels of the Cer species C18:0, C20:0, and C22:0 and a significant decrease in the level of the SM species C25:1 and C26:1 [257]. The same study showed that chronic palmitate exposure reduced SM and Chol content in the endoplasmic reticulum and disrupted its lipid rafts, supporting the hypothesis suggesting that disruption in endoplasmic reticulum-to-Golgi apparatus trafficking leads to protein overload and induces endoplasmic reticulum stress.

### Lipidome dynamics in cancer cells

Neoplastic cells exhibit altered lipid metabolism, impacting the membrane lipidome, which allows these cells to withstand immune system responses. Studies on normal colon cells and four colon cancer cell types revealed distinct lipidomic signatures in both the membrane lipidome and their extracellular vesicles between the healthy and cancer cells [258]. All cancer cells showed an altered profile in glycerophospholipids containing ethanolamine, diacyl-PC, diacyl-PE or PE plasmalogen [258]. Moreover, a study on melanoma revealed that melanoma cells with low metastatic potential are enriched in PIs with saturated and short fatty acid tails compared to those with high metastatic potential, while the membranes of melanoma cell-derived exosomes contained higher amounts of LPC, PA and SM compared to the cells from which they originated [259]. Furthermore, a distinct tumor subtype-specific lipidome signature has also reported for breast cancer cells [260].

Although this section describes only a few examples, it is clear that changes in the lipidomic landscape of cancer cell membranes and tumor cell-derived extracellular vesicles may lead to the discovery of promising biomarkers for designing novel therapeutic and diagnostic techniques.

## Final remarks

The variability in the lipid profile between different cell types and/or the distribution of lipid species within subcellular organelles is poorly understood. To date, only a few comparative studies have provided information on the amount of lipids in different organelles, and therefore, to gain a better understanding of the subcellular lipidome, future quantitative comparative lipidomic studies are essential. In addition, changes in the cellular and organelle lipid profile over time or in response to either physiological or pathological stimuli make this task even more challenging. Another extremely complex issue for which new solutions are urgently needed is related to the annotation of lipids and the management of data pertaining to the curation of lipid structures in subcellular organelles. MS is an indispensable tool for lipidomic analysis and it should be performed with sample that have been efficiently extracted and separated. Obtaining pure lipid samples specific to each organelle is a prerequisite for accurate mapping of subcellular lipidomes, and the extraction methods available today need to be improved to enhance the purity of different subcellular fractions. In addition, without the ability to separate the most abundant lipid species, it is challenging to provide accurate identification of lipids or quantification data on lipids in low abundance. The resolution of this problem largely depends on instrument capabilities and sensitivities, highlighting the need for further development of MS, which may increase the understanding of lipidome variabilities and perturbations in health and disease and in elucidating the cellular functions of different lipid species.

## Data Availability

No new data were created in this study. No data availability.

## Abbreviations

BMP: Bis(monoacylglyceryl)phosphate
Cer: Ceramide
Chol: Cholesterol
CL: Cardiolipin
COX: Cyclooxygenase
DG: Diacylglycerol
ESI: Electrospray ionization
FFA: Free fatty acids
GC: Gas chromatography
GC–MS: Gas chromatography coupled to mass spectrometry
GSL: Glycosphingolipids
HexCer: Hexosylceramide
HPTLC: High performance thin layer chromatography
HRMS: High-resolution mass spectrometry
LC: Liquid chromatography
LC–MS: Liquid chromatography coupled to mass spectrometry
LC–MS/MS: Chromatography with tandem mass spectrometry
LOX: Lipoxygenase
LPC: Lysophosphatidylcholine
LPE: Lysophosphatidylethanolamine
LPG: Lysophosphatidylglycerol
LPI: Lysophosphatidylinositol
LPS: Lysophosphatidylserine
MG: Monoacylglycerol
MS: Mass spectrometry
MUFA: Monounsaturated fatty acids
MVB: Multivesicular bodies
NPC1: Niemann-Pick type C1
OH-Cer: Hydroxylated ceramide
OH-HexCer: Hydroxylated hexosylceramide
PA: Phosphatidic acid
PC: Phosphatidylcholine
PE: Phosphatidylethanolamine
PG: Phosphatidylglycerol
PI: Phosphatidylinositol
PI3P: Phosphatidylinositol 3-phosphate
PI(3,5): P_2_, phosphatidylinositol 3,5-bisphosphate
PI(3,4,5): P_3_, phosphatidylinositol (3,4,5)-trisphosphate
PI4P: Phosphatidylinositol 4-phosphate
PI(4,5): P_2_, phosphatidylinositol 4,5-bisphosphate
PLA_2_: Phospholipase 2
PS: Phosphatidylserine
PUFA: Polyunsaturated fatty acids
QIT: Quadrupole ion trap
ROS: Reactive oxygen species
SFA: Saturated fatty acids
SM: Sphingomyelin
TG: Triacylglycerol
TLC: Thin layer chromatography
TOF: Time of flight
